# 2-({4-[4-(1*H*-Benzimidazol-2-yl)phen­yl]-1*H*-1,2,3-triazol-1-yl}meth­oxy)ethanol

**DOI:** 10.1107/S1600536812023410

**Published:** 2012-05-26

**Authors:** Abdelaaziz Ouahrouch, Moha Taourirte, Hassan B. Lazrek, Jan W. Bats, Joachim W. Engels

**Affiliations:** a Laboratory of Bioorganic and Macromolecular Chemistry, Department of Chemistry, Faculty of Sciences and Technology Guéliz (FSTG), BP 549, Marrakech, Morocco; b Laboratory of Biomolecular and Medicinal Chemistry, Department of Chemistry, Faculty of Sciences Semlalia, Marrakech, Morocco; c Institut für Organische Chemie, Universität Frankfurt, Max-von-Laue-Strasse 7, D-60438 Frankfurt am Main, Germany

## Abstract

In the title molecule, C_18_H_17_N_5_O_2_, the dihedral angle between the benzene plane and the benzimidazole plane is 19.8 (1)° and the angle between the benzene plane and the triazole plane is 16.7 (1)°. In the crystal, mol­ecules are connected by O—H⋯N hydrogen bonds, forming zigzag chains along the *c*-axis direction. The chains are connected by bifurcated N—H⋯(N,N) hydrogen bonds into layers parallel to (100). These layers are connected along the *a*-axis direction by weak C—H⋯O contacts, forming a three-dimensional network.

## Related literature

For the bioactivity of benzimidazoles, see: Tebbe *et al.* (1997 ▶); Andrzejewska *et al.* (2002 ▶); Navarrete-Vázquez *et al.* (2003 ▶); Terzioglu *et al.* (2004 ▶); Özden *et al.* (2005 ▶). For the bioactivity of 1,2,3-triazoles, see: Chen *et al.* (2000 ▶); Manfredini *et al.* (2000 ▶). For the synthetic methods, see: Huisgen (1963 ▶); Crisp & Flynn (1993 ▶); Wu *et al.* (2004 ▶); Navarrete-Vázquez *et al.* (2007 ▶); Krim *et al.* (2009 ▶).
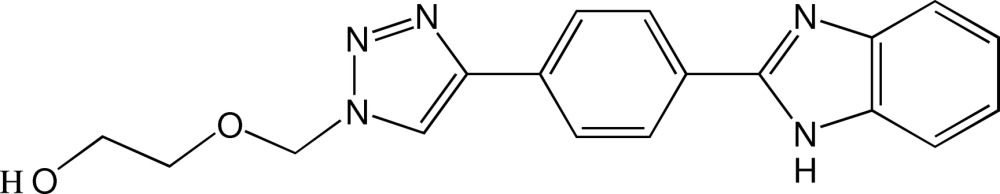


## Experimental

### 

#### Crystal data

C_18_H_17_N_5_O_2_
*M* *_r_* = 335.37
Monoclinic, 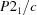

*a* = 10.4449 (5) Å
*b* = 7.7754 (4) Å
*c* = 20.2119 (10) Å
β = 99.354 (1)°
*V* = 1619.65 (14) Å^3^
*Z* = 4
Mo *K*α radiation
μ = 0.09 mm^−1^
*T* = 170 K
0.40 × 0.32 × 0.11 mm

#### Data collection

Siemens SMART 1K CCD diffractometer
Absorption correction: multi-scan (*SADABS*; Sheldrick, 2000 ▶) *T*_min_ = 0.953, *T*_max_ = 0.990
15768 measured reflections
3295 independent reflections
2562 reflections with *I* > 2σ(*I*)
*R*_int_ = 0.047

#### Refinement

*R*[*F* ^2^ > 2σ(*F* ^2^)] = 0.059
*wR*(*F* ^2^) = 0.102
*S* = 1.14
3295 reflections
235 parameters
H atoms treated by a mixture of independent and constrained refinement
Δρ_max_ = 0.20 e Å^−3^
Δρ_min_ = −0.19 e Å^−3^

### 

Data collection: *SMART* (Siemens, 1995 ▶); cell refinement: *SAINT* (Siemens, 1995 ▶); data reduction: *SAINT*; program(s) used to solve structure: *SHELXS97* (Sheldrick, 2008 ▶); program(s) used to refine structure: *SHELXL97* (Sheldrick, 2008 ▶); molecular graphics: *SHELXTL* (Sheldrick, 2008 ▶); software used to prepare material for publication: *SHELXL97*.

## Supplementary Material

Crystal structure: contains datablock(s) global, I. DOI: 10.1107/S1600536812023410/nc2282sup1.cif

Structure factors: contains datablock(s) I. DOI: 10.1107/S1600536812023410/nc2282Isup2.hkl

Supplementary material file. DOI: 10.1107/S1600536812023410/nc2282Isup3.cml

Additional supplementary materials:  crystallographic information; 3D view; checkCIF report

## Figures and Tables

**Table 1:** Hydrogen-bond geometry (Å, °)

*D*—H⋯*A*	*D*—H	H⋯*A*	*D*⋯*A*	*D*—H⋯*A*
N1—H1*A*⋯N4^i^	0.89 (2)	2.50 (2)	3.244 (2)	141.7 (17)
N1—H1*A*⋯N5^i^	0.89 (2)	2.21 (2)	3.088 (2)	170.4 (19)
O2—H2*A*⋯N2^ii^	0.92 (3)	1.85 (3)	2.761 (2)	171 (2)
C15—H15*A*⋯O2^iii^	0.95	2.54	3.271 (2)	134
C16—H16*A*⋯O2^iii^	0.99	2.54	3.258 (2)	129
C17—H17*B*⋯O1^iv^	0.99	2.35	3.279 (2)	155

Symmetry codes: (i) 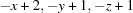; (ii) 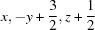; (iii) 
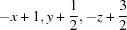
; (iv) 
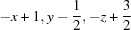
.

**Fig. 1.**
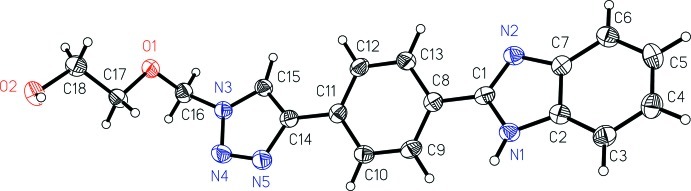
The structure of the title compound, showing the atom-labelling scheme. Displacement ellipsoids are drawn at the 50% probability level. H atoms are drawn as small spheres of an arbitrary radius.

**Fig. 2.**
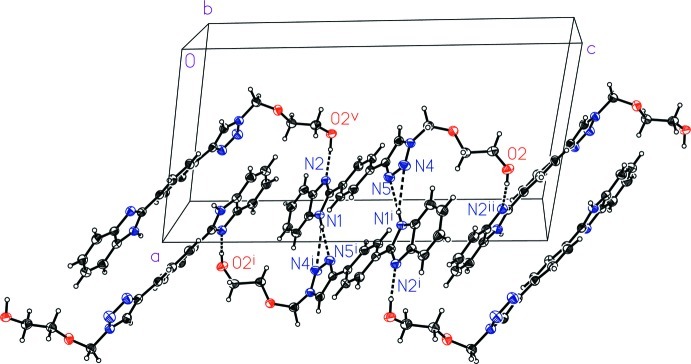
A view of the hydrogen bonding network of the title compound. Displacement ellipsoids are drawn at the 50% probabilty level. Intermolecular hydrogen bonds are shown as dashed lines. The symmetry codes are: (i) 2 - x, 1 - y, 1 - z; (ii) x, 3/2 - y, 1/2 + z; (v) x, 3/2 - y, -1/2 + z.

## supplementary crystallographic information

### Comment

Benzimidazoles are important pharmacophores in modern drug discovery (Tebbe *et al.*, 1997). They are present in various bioactive compounds, possessing, *e.g.* antiparasitic (Navarrete-Vázquez *et al.*, 2003), antimicrobial (Özden *et al.*, 2005), antihistaminic (Terzioglu *et al.*, 2004) and antitumor (Andrzejewska *et al.*, 2002) activities. In addition, 1,2,3-triazoles are potent antibacterial (Chen *et al.*, 2000) and antiproliferative (Manfredini *et al.*, 2000) agents. The most widely used method for their synthesis is the Huisgen 1,3-dipolar cycloaddition of alkynes with organic azides (Huisgen, 1963). Copper-catalyzed click chemistry, involving azides and terminal acetylenes, has enabled practical and efficient preparation of 1,4-disubstituted 1,2,3-triazoles, from a wide range of substrates with excellent selectivity (Wu *et al.*, 2004). In connection to our previous studies on the synthesis of acyclonucleosides (Krim *et al.*, 2009), we decided to explore the feasibility of the click chemistry for the synthesis of novel 1,2,3-triazoles containing a benzimidazole moiety coupled *via* a benzene ring. Thus the title compound was prepared and its crystal structure is reported herein.

A view of the molecular structure of the title compound is shown in Fig. 1. The molecule contains three planar parts: the benzimidazole, the benzene and the triazole groups. The angle between the benzene and benzimidazole planes is 19.8 (1)°, while the angle between the benzene and triazole planes is 16.7 (1)°. The (2-hydroxyethoxy)methyl group points away from the triazole plane [torsion angle N4—N3—C16—O1: 88.6 (2)°]. The C16—O1 bond has a *gauche* conformation, while the O1—C17 and the C17—C18 bonds have *trans* conformations.

The molecules of the title compound are connected by intermolecular O—H···N hydrogen bonds to form zigzag chains along the *c* axis direction (Fig.2, Table 1). Adjacent molecules in each chain are related by c-gilde plane symmetry. Neighboring chains are connected by intermolecular N—H···N hydrogen bonds between imidazole and triazole groups to form layers parallel to (1 0 0). The N—H···N hydrogen bond is bifurcated with both atoms N4 and N5 acting as acceptor atoms. There are two symmetry-related N—H···N bonds between each pair of molecules. The hydrogen bonded layers are connected along the *a* axis direction by additional intermolecular weak C—H···O contacts to form a three-dimensional framework.

### Experimental

The title compound has been prepared in four steps starting from 4-[(trimethylsilyl)ethynyl]benzaldehyde. The starting material was reacted with benzimidazole in the presence of sodium metabisulfite using microwave irradiation (Navarrete-Vázquez *et al.*, 2007). The resulting product was deprotected with tetrabutylammonium fluoride in tetrahydrofuran to 2-(4-ethynylphenyl)-1*H*-benzimidazole (Crisp & Flynn, 1993). Cycloaddition of the latter product with [(2-acetoxyethoxy)methyl]azide in the presence of CuI, followed by deprotection of the acetyl group (Krim *et al.*, 2009), afforded the title compound in good yield. The crude product was purified by passing through a column packed with silica gel. Single crystals suitable for X-ray analysis were obtained by slow recrystallizion from ethanol. The melting point is approximately 502–504 K.

### Refinement

The H atoms on C atoms were positioned geometrically and treated as riding: C_planar_—H=0.95 Å, C_methylene_—H=0.99 Å, *U*_iso_(H)=1.2*U*_eq_(parent C-atom). The H atoms on the N and O atoms were taken from a difference Fourier synthesis and were refined.

### Crystal data

**Table:**

C_18_H_17_N_5_O_2_	*F*(000) = 704
*M**_r_* = 335.37	*D*_x_ = 1.375 Mg m^−^^3^
Monoclinic, *P*2_1_/*c*	Mo *K*α radiation, λ = 0.71073 Å
Hall symbol: -P 2ybc	Cell parameters from 8027 reflections
*a* = 10.4449 (5) Å	θ = 3–24°
*b* = 7.7754 (4) Å	µ = 0.09 mm^−^^1^
*c* = 20.2119 (10) Å	*T* = 170 K
β = 99.354 (1)°	Block, yellow
*V* = 1619.65 (14) Å^3^	0.40 × 0.32 × 0.11 mm
*Z* = 4	

### Data collection

**Table:**

Siemens SMART 1K CCD diffractometer	3295 independent reflections
Radiation source: normal-focus sealed tube	2562 reflections with *I* > 2σ(*I*)
Graphite monochromator	*R*_int_ = 0.047
ω scans	θ_max_ = 26.8°, θ_min_ = 2.0°
Absorption correction: multi-scan (*SADABS*; Sheldrick, 2000)	*h* = −13→13
*T*_min_ = 0.953, *T*_max_ = 0.990	*k* = −9→9
15768 measured reflections	*l* = −25→25

### Refinement

**Table:**

Refinement on *F*^2^	Secondary atom site location: difference Fourier map
Least-squares matrix: full	Hydrogen site location: inferred from neighbouring sites
*R*[*F*^2^ > 2σ(*F*^2^)] = 0.059	H atoms treated by a mixture of independent and constrained refinement
*wR*(*F*^2^) = 0.102	*w* = 1/[σ^2^(*F*_o_^2^) + (0.023*P*)^2^ + 0.6*P*] where *P* = (*F*_o_^2^ + 2*F*_c_^2^)/3
*S* = 1.14	(Δ/σ)_max_ = 0.001
3295 reflections	Δρ_max_ = 0.20 e Å^−^^3^
235 parameters	Δρ_min_ = −0.19 e Å^−^^3^
0 restraints	Extinction correction: *SHELXL97* (Sheldrick, 2008), Fc^*^=kFc[1+0.001xFc^2^λ^3^/sin(2θ)]^-1/4^
Primary atom site location: structure-invariant direct methods	Extinction coefficient: 0.0056 (7)

### Special details

**Table:**

Geometry. All e.s.d.'s (except the e.s.d. in the dihedral angle between two l.s. planes) are estimated using the full covariance matrix. The cell e.s.d.'s are taken into account individually in the estimation of e.s.d.'s in distances, angles and torsion angles; correlations between e.s.d.'s in cell parameters are only used when they are defined by crystal symmetry. An approximate (isotropic) treatment of cell e.s.d.'s is used for estimating e.s.d.'s involving l.s. planes.
Refinement. Refinement of *F*^2^ against ALL reflections. The weighted *R*-factor *wR* and goodness of fit *S* are based on *F*^2^, conventional *R*-factors *R* are based on *F*, with *F* set to zero for negative *F*^2^. The threshold expression of *F*^2^ > σ(*F*^2^) is used only for calculating *R*-factors(gt) *etc*. and is not relevant to the choice of reflections for refinement. *R*-factors based on *F*^2^ are statistically about twice as large as those based on *F*, and *R*- factors based on ALL data will be even larger.

### Fractional atomic coordinates and isotropic or equivalent isotropic displacement parameters (Å^2^)

**Table:**

	*x*	*y*	*z*	*U*_iso_*/*U*_eq_	
O1	0.50237 (13)	0.39456 (17)	0.71767 (6)	0.0308 (3)	
O2	0.67189 (14)	0.21899 (18)	0.87378 (7)	0.0335 (4)	
N1	1.07016 (15)	0.9376 (2)	0.37941 (8)	0.0265 (4)	
N2	0.91403 (15)	1.1341 (2)	0.37934 (8)	0.0277 (4)	
N3	0.56182 (15)	0.3846 (2)	0.60950 (7)	0.0272 (4)	
N4	0.65832 (16)	0.2730 (2)	0.60375 (8)	0.0350 (4)	
N5	0.73569 (16)	0.3493 (2)	0.56742 (8)	0.0331 (4)	
C1	0.95442 (18)	0.9778 (2)	0.39886 (9)	0.0252 (4)	
C2	1.10804 (18)	1.0777 (2)	0.34516 (9)	0.0265 (4)	
C3	1.21525 (18)	1.1085 (3)	0.31418 (10)	0.0317 (5)	
H3A	1.2823	1.0256	0.3148	0.038*	
C4	1.2198 (2)	1.2654 (3)	0.28242 (10)	0.0350 (5)	
H4A	1.2918	1.2912	0.2608	0.042*	
C5	1.12061 (19)	1.3873 (3)	0.28139 (10)	0.0343 (5)	
H5A	1.1267	1.4936	0.2589	0.041*	
C6	1.01424 (19)	1.3565 (3)	0.31228 (9)	0.0310 (5)	
H6A	0.9471	1.4393	0.3112	0.037*	
C7	1.00880 (17)	1.1996 (2)	0.34504 (9)	0.0257 (4)	
C8	0.88408 (17)	0.8577 (2)	0.43576 (9)	0.0253 (4)	
C9	0.91089 (18)	0.6817 (2)	0.43692 (9)	0.0283 (5)	
H9A	0.9749	0.6389	0.4127	0.034*	
C10	0.84603 (18)	0.5686 (2)	0.47263 (9)	0.0268 (4)	
H10A	0.8647	0.4491	0.4721	0.032*	
C11	0.75345 (18)	0.6289 (2)	0.50939 (9)	0.0258 (4)	
C12	0.72431 (18)	0.8045 (3)	0.50722 (9)	0.0288 (5)	
H12A	0.6595	0.8469	0.5310	0.035*	
C13	0.78828 (18)	0.9175 (3)	0.47105 (9)	0.0289 (5)	
H13A	0.7671	1.0364	0.4701	0.035*	
C14	0.68875 (18)	0.5107 (2)	0.55019 (9)	0.0256 (4)	
C15	0.57733 (18)	0.5326 (2)	0.57719 (9)	0.0265 (4)	
H15A	0.5229	0.6312	0.5738	0.032*	
C16	0.46450 (19)	0.3426 (3)	0.65116 (9)	0.0302 (5)	
H16A	0.3817	0.3995	0.6326	0.036*	
H16B	0.4495	0.2168	0.6499	0.036*	
C17	0.59651 (18)	0.2857 (2)	0.75702 (9)	0.0288 (4)	
H17A	0.6829	0.3018	0.7438	0.035*	
H17B	0.5713	0.1634	0.7505	0.035*	
C18	0.59977 (19)	0.3374 (3)	0.82907 (10)	0.0334 (5)	
H18A	0.6391	0.4531	0.8364	0.040*	
H18B	0.5099	0.3439	0.8387	0.040*	
H1A	1.118 (2)	0.846 (3)	0.3930 (10)	0.040 (6)*	
H2A	0.756 (3)	0.258 (3)	0.8779 (12)	0.068 (8)*	

### Atomic displacement parameters (Å^2^)

**Table:**

	*U*^11^	*U*^22^	*U*^33^	*U*^12^	*U*^13^	*U*^23^
O1	0.0328 (8)	0.0313 (8)	0.0286 (7)	0.0077 (6)	0.0061 (6)	0.0048 (6)
O2	0.0256 (8)	0.0346 (8)	0.0382 (8)	−0.0039 (7)	−0.0008 (6)	0.0083 (6)
N1	0.0222 (9)	0.0261 (9)	0.0313 (9)	0.0041 (7)	0.0043 (7)	0.0013 (7)
N2	0.0257 (9)	0.0280 (9)	0.0289 (9)	0.0042 (7)	0.0026 (7)	−0.0002 (7)
N3	0.0248 (9)	0.0285 (9)	0.0286 (9)	0.0033 (7)	0.0053 (7)	0.0029 (7)
N4	0.0326 (10)	0.0313 (10)	0.0427 (10)	0.0087 (8)	0.0114 (8)	0.0057 (8)
N5	0.0304 (9)	0.0340 (10)	0.0362 (9)	0.0060 (8)	0.0096 (8)	0.0050 (8)
C1	0.0226 (10)	0.0288 (11)	0.0234 (10)	0.0030 (8)	0.0015 (8)	−0.0039 (8)
C2	0.0250 (10)	0.0266 (10)	0.0267 (10)	0.0009 (8)	0.0002 (8)	−0.0009 (8)
C3	0.0246 (11)	0.0352 (12)	0.0357 (11)	0.0023 (9)	0.0063 (9)	−0.0009 (9)
C4	0.0291 (11)	0.0408 (13)	0.0358 (11)	−0.0034 (10)	0.0073 (9)	0.0024 (9)
C5	0.0342 (12)	0.0337 (12)	0.0334 (11)	−0.0038 (10)	0.0003 (9)	0.0079 (9)
C6	0.0292 (11)	0.0302 (11)	0.0316 (11)	0.0036 (9)	−0.0007 (9)	0.0012 (9)
C7	0.0224 (10)	0.0289 (11)	0.0251 (10)	−0.0009 (8)	0.0017 (8)	−0.0018 (8)
C8	0.0226 (10)	0.0296 (11)	0.0225 (9)	0.0023 (8)	0.0000 (8)	−0.0002 (8)
C9	0.0222 (10)	0.0340 (12)	0.0287 (10)	0.0040 (9)	0.0041 (8)	−0.0043 (9)
C10	0.0244 (10)	0.0265 (10)	0.0289 (10)	0.0029 (8)	0.0020 (8)	−0.0011 (8)
C11	0.0233 (10)	0.0315 (11)	0.0215 (9)	0.0014 (8)	0.0003 (8)	0.0002 (8)
C12	0.0274 (11)	0.0338 (11)	0.0262 (10)	0.0061 (9)	0.0075 (8)	−0.0007 (9)
C13	0.0313 (11)	0.0270 (11)	0.0282 (10)	0.0059 (9)	0.0043 (9)	0.0020 (8)
C14	0.0248 (10)	0.0277 (10)	0.0229 (10)	0.0040 (8)	0.0000 (8)	−0.0013 (8)
C15	0.0280 (11)	0.0258 (11)	0.0250 (10)	0.0036 (8)	0.0019 (8)	0.0005 (8)
C16	0.0259 (10)	0.0335 (11)	0.0318 (11)	0.0004 (9)	0.0063 (9)	0.0048 (9)
C17	0.0271 (11)	0.0250 (10)	0.0344 (11)	0.0022 (9)	0.0054 (9)	0.0051 (8)
C18	0.0266 (11)	0.0368 (12)	0.0360 (11)	0.0048 (9)	0.0019 (9)	0.0020 (9)

### Geometric parameters (Å, º)

**Table:**

O1—C16	1.398 (2)	C6—C7	1.394 (3)
O1—C17	1.436 (2)	C6—H6A	0.9500
O2—C18	1.418 (2)	C8—C9	1.396 (3)
O2—H2A	0.92 (3)	C8—C13	1.399 (3)
N1—C1	1.367 (2)	C9—C10	1.383 (3)
N1—C2	1.382 (2)	C9—H9A	0.9500
N1—H1A	0.89 (2)	C10—C11	1.393 (3)
N2—C1	1.325 (2)	C10—H10A	0.9500
N2—C7	1.394 (2)	C11—C12	1.398 (3)
N3—C15	1.346 (2)	C11—C14	1.471 (3)
N3—N4	1.349 (2)	C12—C13	1.382 (3)
N3—C16	1.458 (2)	C12—H12A	0.9500
N4—N5	1.318 (2)	C13—H13A	0.9500
N5—C14	1.371 (2)	C14—C15	1.374 (3)
C1—C8	1.465 (3)	C15—H15A	0.9500
C2—C3	1.390 (3)	C16—H16A	0.9900
C2—C7	1.404 (3)	C16—H16B	0.9900
C3—C4	1.383 (3)	C17—C18	1.506 (3)
C3—H3A	0.9500	C17—H17A	0.9900
C4—C5	1.402 (3)	C17—H17B	0.9900
C4—H4A	0.9500	C18—H18A	0.9900
C5—C6	1.381 (3)	C18—H18B	0.9900
C5—H5A	0.9500		
			
C16—O1—C17	115.06 (14)	C8—C9—H9A	119.4
C18—O2—H2A	104.2 (16)	C9—C10—C11	120.37 (18)
C1—N1—C2	107.60 (16)	C9—C10—H10A	119.8
C1—N1—H1A	125.3 (13)	C11—C10—H10A	119.8
C2—N1—H1A	126.2 (14)	C10—C11—C12	118.60 (18)
C1—N2—C7	105.41 (15)	C10—C11—C14	120.69 (17)
C15—N3—N4	110.95 (15)	C12—C11—C14	120.70 (17)
C15—N3—C16	128.43 (16)	C13—C12—C11	121.04 (18)
N4—N3—C16	120.43 (15)	C13—C12—H12A	119.5
N5—N4—N3	107.03 (15)	C11—C12—H12A	119.5
N4—N5—C14	109.09 (15)	C12—C13—C8	120.37 (18)
N2—C1—N1	112.19 (17)	C12—C13—H13A	119.8
N2—C1—C8	125.05 (17)	C8—C13—H13A	119.8
N1—C1—C8	122.75 (17)	N5—C14—C15	107.68 (17)
N1—C2—C3	132.73 (18)	N5—C14—C11	122.44 (17)
N1—C2—C7	105.15 (16)	C15—C14—C11	129.88 (17)
C3—C2—C7	122.11 (18)	N3—C15—C14	105.25 (17)
C4—C3—C2	116.87 (19)	N3—C15—H15A	127.4
C4—C3—H3A	121.6	C14—C15—H15A	127.4
C2—C3—H3A	121.6	O1—C16—N3	112.07 (15)
C3—C4—C5	121.50 (19)	O1—C16—H16A	109.2
C3—C4—H4A	119.3	N3—C16—H16A	109.2
C5—C4—H4A	119.3	O1—C16—H16B	109.2
C6—C5—C4	121.51 (19)	N3—C16—H16B	109.2
C6—C5—H5A	119.2	H16A—C16—H16B	107.9
C4—C5—H5A	119.2	O1—C17—C18	106.53 (15)
C5—C6—C7	117.71 (19)	O1—C17—H17A	110.4
C5—C6—H6A	121.1	C18—C17—H17A	110.4
C7—C6—H6A	121.1	O1—C17—H17B	110.4
C6—C7—N2	130.04 (18)	C18—C17—H17B	110.4
C6—C7—C2	120.30 (18)	H17A—C17—H17B	108.6
N2—C7—C2	109.64 (16)	O2—C18—C17	111.68 (16)
C9—C8—C13	118.37 (17)	O2—C18—H18A	109.3
C9—C8—C1	121.16 (17)	C17—C18—H18A	109.3
C13—C8—C1	120.47 (17)	O2—C18—H18B	109.3
C10—C9—C8	121.20 (18)	C17—C18—H18B	109.3
C10—C9—H9A	119.4	H18A—C18—H18B	107.9
			
C15—N3—N4—N5	−0.3 (2)	C13—C8—C9—C10	−0.8 (3)
C16—N3—N4—N5	−175.62 (15)	C1—C8—C9—C10	178.93 (16)
N3—N4—N5—C14	0.3 (2)	C8—C9—C10—C11	−1.1 (3)
C7—N2—C1—N1	0.1 (2)	C9—C10—C11—C12	2.4 (3)
C7—N2—C1—C8	−178.63 (17)	C9—C10—C11—C14	−176.93 (17)
C2—N1—C1—N2	0.3 (2)	C10—C11—C12—C13	−1.9 (3)
C2—N1—C1—C8	179.09 (16)	C14—C11—C12—C13	177.50 (17)
C1—N1—C2—C3	−179.47 (19)	C11—C12—C13—C8	0.0 (3)
C1—N1—C2—C7	−0.60 (19)	C9—C8—C13—C12	1.4 (3)
N1—C2—C3—C4	178.25 (19)	C1—C8—C13—C12	−178.36 (17)
C7—C2—C3—C4	−0.5 (3)	N4—N5—C14—C15	−0.3 (2)
C2—C3—C4—C5	−0.2 (3)	N4—N5—C14—C11	−179.91 (16)
C3—C4—C5—C6	0.3 (3)	C10—C11—C14—N5	16.1 (3)
C4—C5—C6—C7	0.3 (3)	C12—C11—C14—N5	−163.22 (17)
C5—C6—C7—N2	−179.28 (18)	C10—C11—C14—C15	−163.43 (18)
C5—C6—C7—C2	−1.0 (3)	C12—C11—C14—C15	17.2 (3)
C1—N2—C7—C6	177.95 (19)	N4—N3—C15—C14	0.1 (2)
C1—N2—C7—C2	−0.5 (2)	C16—N3—C15—C14	174.99 (17)
N1—C2—C7—C6	−177.94 (16)	N5—C14—C15—N3	0.1 (2)
C3—C2—C7—C6	1.1 (3)	C11—C14—C15—N3	179.71 (18)
N1—C2—C7—N2	0.7 (2)	C17—O1—C16—N3	−77.18 (19)
C3—C2—C7—N2	179.71 (16)	C15—N3—C16—O1	−85.9 (2)
N2—C1—C8—C9	160.37 (18)	N4—N3—C16—O1	88.6 (2)
N1—C1—C8—C9	−18.3 (3)	C16—O1—C17—C18	−166.70 (15)
N2—C1—C8—C13	−19.9 (3)	O1—C17—C18—O2	169.44 (15)
N1—C1—C8—C13	161.50 (17)		

### Hydrogen-bond geometry (Å, º)

**Table:**

*D*—H···*A*	*D*—H	H···*A*	*D*···*A*	*D*—H···*A*
N1—H1*A*···N4^i^	0.89 (2)	2.50 (2)	3.244 (2)	141.7 (17)
N1—H1*A*···N5^i^	0.89 (2)	2.21 (2)	3.088 (2)	170.4 (19)
O2—H2*A*···N2^ii^	0.92 (3)	1.85 (3)	2.761 (2)	171 (2)
C15—H15*A*···O2^iii^	0.95	2.54	3.271 (2)	134
C16—H16*A*···O2^iii^	0.99	2.54	3.258 (2)	129
C17—H17*B*···O1^iv^	0.99	2.35	3.279 (2)	155

Symmetry codes: (i) −*x*+2, −*y*+1, −*z*+1; (ii) *x*, −*y*+3/2, *z*+1/2; (iii) −*x*+1, *y*+1/2, −*z*+3/2; (iv) −*x*+1, *y*−1/2, −*z*+3/2.
